# Identification of KIAA1199 as a Biomarker for Pancreatic Intraepithelial Neoplasia

**DOI:** 10.1038/srep38273

**Published:** 2016-12-06

**Authors:** Han Na Suh, Sohee Jun, Ah-Young Oh, Mrinal Srivastava, Sunhye Lee, Cullen M. Taniguchi, Songlin Zhang, Won Sup Lee, Junjie Chen, Bum-Joon Park, Jae-Il Park

**Affiliations:** 1 Department of Experimental Radiation Oncology, The University of Texas MD Anderson Cancer Center, Houston, TX, 77030, USA; 2 Department of Molecular Biology, Pusan National University, Busan, Republic of Korea; 3 Department of Pathology, The University of Texas Medical School, Houston, TX, 77030, USA; 4 Department of Internal Medicine, Gyeong-Sang National University Hospital, Jinju, Republic of Korea; 5 Graduate School of Biomedical Sciences at Houston, Houston, TX, 77030, USA; 6 Program in Cancer Biology, The University of Texas MD Anderson Cancer Center, Houston, TX, 77030, USA

## Abstract

Pancreatic cancer is one of the most aggressive cancers and has an extremely poor prognosis. Despite recent progress in both basic and clinical research, most pancreatic cancers are detected at an incurable stage owing to the absence of disease-specific symptoms. Thus, developing novel approaches for detecting pancreatic cancer at an early stage is imperative. Our in silico and immunohistochemical analyses showed that KIAA1199 is specifically expressed in human pancreatic cancer cells and pancreatic intraepithelial neoplasia, the early lesion of pancreatic cancer, in a genetically engineered mouse model and in human patient samples. We also detected secreted KIAA1199 protein in blood samples obtained from pancreatic cancer mouse models, but not in normal mice. Furthermore, we found that assessing KIAA1199 autoantibody increased the sensitivity of detecting pancreatic cancer. These results indicate the potential benefits of using KIAA1199 as a biomarker for early-stage pancreatic cancer.

Pancreatic cancer is one of the most aggressive cancers and has extremely poor prognosis^1^. Pancreatic cancer cells tend to rapidly metastasize to the lymphatic system and other distant organs. Owing to the absence of disease-specific symptoms for pancreatic cancer, most detected pancreatic cancers are incurable. In spite of the recent progress made in both basic and clinical research, there has been a spike in the mortality rate of pancreatic cancer. To date, the 5-year survival rate of pancreatic cancer patients is about 5%^2^,^3^.

Despite the ambiguity in the panoramic view of human pancreatic ductal adenocarcinoma (PDAC) pathology, accumulating evidence suggests that successive genetic mutations initiate pancreatic cancer^4^,^5^. Recent studies proposed that PDAC might arise from pre-neoplastic lesions including pancreatic intraepithelial neoplasias (PanINs)^6^, mucinous cystic neoplasms, or intraductal papillary mucinous neoplasms^7^,^8^. Given that PanINs exhibit a duct-like characteristic with expression of ductal genes, it was previously considered that PanINs are developed from pancreatic duct. However, mouse models showed that K-Ras oncogenic activation in ductal cells is insufficient to develop PanINs. Instead, recent mouse studies showed that PanINs are developed from the acinar cells via acinar-to-ductal metaplasia^9^,^10^,^11^. Moreover, pancreatobiliary duct cells also exist in PanINs as tumor-initiating cells^12^,^13^. *K-Ras* is frequently mutated in pancreatic cancer (>90%)^14^, resulting in hyperactivation of MAPK signaling and subsequent cell hyperproliferation. In mouse models, *K-Ras*^*G12D*^ oncogenic mutation initiates PanINs^6^, recapitulating the critical role of *K-Ras* in the development of human PDAC. Additionally, *CDKN2A* encoding p16 and *TP53* are frequently mutated in pancreatic cancer (>50% and 60–70%, respectively), leading to an uncontrolled cell cycle and bypass of DNA damage repair^15^. Moreover, mutational inactivation of Smad4 (*DPC4*) contributes to invasive pancreatic cancer^16^,^17^,^18^.

Carbohydrate antigen 19-9 (CA19-9) is a pancreatic cancer biomarker not only for diagnosis but also for assessment of therapy resistance and prognosis^19^,^20^,^21^. However, false-negative results in the Lewis blood type α-β- population^22^ and false-positive results in patients with obstructive jaundice^23^ limit the specificity of CA19-9 to pancreatic cancer. Recently, circulating exosome was also suggested as a biomarker for pancreatic cancer^24^. However, more specific and efficient biomarkers are needed to assess pancreatic cancer for early diagnosis and prognosis.

*KIAA1199* encodes 1361 amino acids of protein containing the G8 domain, which consists of eight glycines and five β-strand pairs^25^. The function of the G8 domain is unknown. KIAA1199 has an N-terminal signal peptide and signal peptide cleavage site, implying that KIAA1199 could have extracellular secretion or membrane-targeting properties. Several mutations in *KIAA1199* were identified in patients with hearing loss, suggesting that *KIAA1199* plays a role in auditory development^26^. KIAA1199 also contributes to breast cancer cell migration with induction of epithelial-mesenchymal transition via calcium signaling^27^,^28^. KIAA1199 also activates Wnt signaling for colorectal cancer cell proliferation^29^. Additionally, KIAA1199 expression is associated with gastric^30^ and colorectal^31^ tumorigenesis. Moreover, KIAA1199 is a potential biomarker of gastric carcinoma^32^.

In this study, our comprehensive approaches suggest that KIAA1199 is a biomarker for pancreatic cancer. Our results show that KIAA1199 is specifically expressed in the early stages of pancreatic tumorigenesis. We also found that detecting autoantibody against KIAA1199 is a highly sensitive method to detect pancreatic cancer from blood samples from pancreatic cancer mouse models and from human patients with pancreatic cancer.

## Results

### Identification of Genes Specifically Expressed in PDAC

To identify biomarkers for PDAC, we performed in silico gene expression analysis of PDAC cDNA microarray datasets, using the Oncomine database (www.oncomine.org)^33^. We found 21 genes that are significantly and frequently upregulated in two pancreatic cancer gene expression datasets (top 1% ranked genes, fold change >2; P < 0.0001): *ADAM8, AHNAK2, BUB1, C16orf75, CCNB1, CDH3, CLTB, ECT2, FOXL1, KIAA1199, KIF14, KIF4A, MYEOV, NMU, P4HA1, SDR16C5, SERPINB5, SLC6A14, TPX2, TTLL12*, and *WFDC2* (Supplementary Data 1).

**Figure 1:**
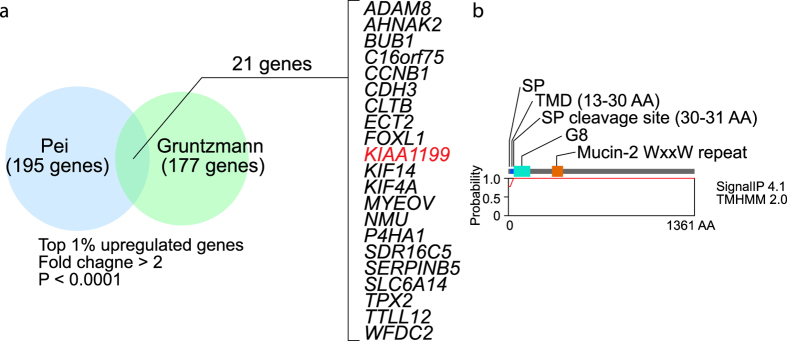
Identification of genes highly expressed in pancreatic ductal adenocarcinoma (PDAC). (**a**) Two cDNA microarray datasets (Pei and Gruntzmann) were analyzed using Oncomine, and 21 genes were selected as the top 1% of upregulated genes (fold change > 2; P < 0.0001). (**b**) Structure of KIAA1199. SP: signal peptide; TMD: transmembrane domain.

Considering the potential benefits of detecting these proteins in blood serum, we focused on secreted proteins and selected *KIAA1199* (Fig. 1a). Transmembrane protein sequence analysis using the TMHMM 2.0 server (http://www.cbs.dtu.dk/services/TMHMM/) showed that KIAA1199 harbors a signal peptide (SP), transmembrane domain, and SP cleavage site (Fig. 1b), implying that KIAA1199 has extracellular secretion or cellular membrane–targeting properties.

### Expression of KIAA1199 in Human PDAC

**Figure 2:**
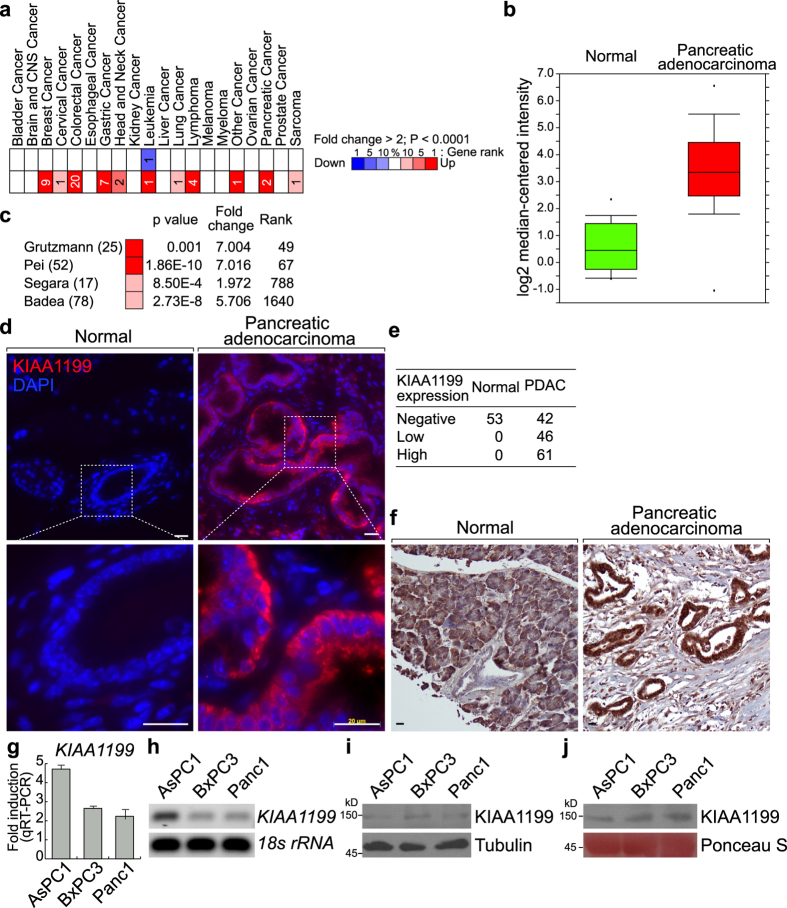
Expression of KIAA1199 in human pancreatic ductal adenocarcinoma (PDAC). (**a**) Expression of KIAA1199 in human cancers via Oncomine analysis. (**b**,**c**) Expression of KIAA1199 in human pancreatic adenocarcinoma via Oncomine analysis. (**d**–**f**) Expression of KIAA1199 in human pancreatic adenocarcinoma. Fluorescence immunohistochemical analysis of KIAA1199 of human pancreatic cancer tissue microarray (TMA; US Biomax PA207), using anti-KIAA1199 antibody (**d**). Quantitative analysis of KIAA1199 expression in TMA (**e**). Chromogenic immunohistochemical analysis of human pancreatic cancer tissue microarray (US Biomax BIC14011a, PA242b) using-KIAA1199 antibody and DAB substrate (**f**). (**g**,**h**) mRNA expression of *KIAA1199* in PDAC cell lines (AsPC-1, BxPC-3, and Panc-1). Quantitative RT-PCR (**g**) and semi-quantitative RT-PCR (**h**). *KIAA1199* expression in PDAC cell lines compared to HPNE (normal pancreas epithelial cell line) and normalized to *18 S rRNA*. Gel images shown have been cropped to show the relevant band. Full-length gels are presented in Supplementary Figure 3. (**i**,**j**) Endogenous and secreted protein expression of KIAA1199 in PDAC cell lines (AsPC-1, BxPC-3, and Panc-1). Molecular weight of KIAA1199 was approximately 150 kDa. Blot images shown have been cropped to show the relevant band. Full-length blots are presented in Supplementary Figure 3.

Next, we analyzed expression of *KIAA1199* in human cancer. *In silico* analysis of Oncomine datasets showed that *KIAA1199* upregulation occurs in various cancers including colorectal cancer, breast cancer, gastric cancer, lymphoma, and pancreatic cancer (Fig. 2a). We also confirmed the upregulation of *KIAA1199* in human PDACs (Fig. 2b and c). To validate the upregulation of KIAA1199 in PDAC, we assessed expression of KIAA1199 protein in PDAC samples. Fluorescent immunohistochemistry results showed that KIAA1199 is only detected in the cytosol of human PDAC cells (71.8%; N = 149) but is undetectable in normal pancreatic cells (0%; N = 53) (Fig. 2d and e). We also confirmed that KIAA1199 expression and histological morphology in PDAC samples using DAB substrate (Fig. 2f, Supplementary Data 2, Supplementary Figure 2).

Additionally, we examined the expression of *KIAA1199* mRNA and protein in human pancreatic cancer cell lines: AsPC-1, BxPC-3, and Panc-1. Pancreatic cancer cell lines showed both transcriptional and protein expression of KIAA1199 (Fig. 2g–i). While AsPC1 showed the higher expression of *KIAA1199* mRNA, the protein level of KIAA1199 was similar among three cell lines, implying the post-transcriptional regulation of KIAA1199 expression. Also, secreted KIAA1199 protein was detected in the conditioned media of PDAC cell lines (Fig. 2j and Supplementary Figure 1). These results suggest that KIAA1199 is upregulated and secreted in PDAC cells but not in normal pancreatic cells.

### Expression of KIAA1199 in Mouse and Human PanINs

**Figure 3:**
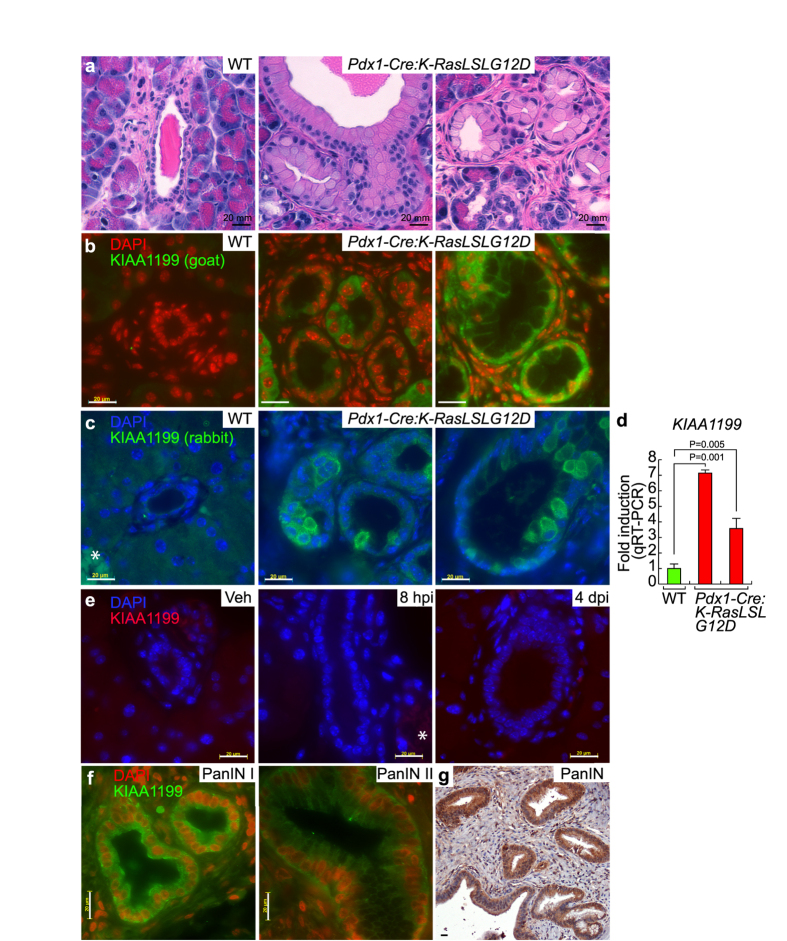
Expression of KIAA1199 in mouse and human pancreatic intraepithelial neoplasias (PanINs). (**a**) Hematoxylin and eosin staining of normal pancreatic and PanIN tissue 7-month-old mice (wild-type [WT; N = 6] vs. *Pdx1-Cre:K-Ras*^*LSLG12D*^ [PanIN; N = 6]). (**b**,**c**) Immunostaining of normal pancreatic and PanIN tissue using anti-KIAA1199 antibodies (goat [b] and rabbit [c]). *Non-specific signal from red blood cells. (**d**) Transcriptional upregulation of *KIAA1199* in mouse PanINs. (qRT-PCR) analysis of normal pancreatic and PanINs cells. (**e**) Immunostaining of normal pancreas (vehicle) and caerulein-induced acute pancreatitis tissue samples. Vehicle (veh) vs. 8 hours after caerulein-induced injury (8 hpi) vs. 4 days after caerulein-induced injury (4 dpi). (**f**,**g**) Immunohistochemical analysis of KIAA1199 of human PanIN grade I and II tissue microarray (TMA; Biomax BIC14011a), using anti-KIAA1199 antibody.

Despite recent advances in pancreatic cancer research, detecting early-stage pancreatic tumors remains elusive. Nonetheless, genetically engineered mouse models have provided valuable information regarding the early events of pancreatic tumorigenesis. To determine whether KIAA1199 is a biomarker of early pancreatic cancer, we investigated whether KIAA1199 expression is also specifically expressed in a pancreatic cancer mouse model. *Pdx1-Cre:K-Ras*^*LSLG12D*^ mice (7-month-old) conditionally express oncogenic *K-Ras (K-Ras*^*G12D*^) in the pancreas and develop PanINs, an early lesion of PDAC (Fig. 3a). Using this mouse model, we immunostained normal pancreatic cells and PanIN tissue using two different type of antibodies. KIAA1199 was not detected in normal pancreatic tissue of wild-type (WT) mice (N = 6) (Fig. 3b and c) but was markedly expressed in the cytosol of ductal epithelial cells of all PanIN mouse models (N = 6) (Fig. 3b and c), which recapitulates the specific expression of KIAA1199 in human PDACs (see Fig. 2d–f).

Notably, the expression pattern of KIAA1199 was heterogeneous in PanIN lesions. For example, KIAA1199 expression was only detectable in some regions of PanINs but not in all lesions of the same mouse. We also found that only some cells of PanIN lesions exhibited KIAA1199 expression (Fig. 3b and c). To confirm the expression of KIAA1199 in PanINs, we also performed quantitative reverse transcription polymerase chain reaction (qRT-PCR). Transcriptional expression analysis of pancreatic lysates (WT and PanINs) showed strong expression of *KIAA1199* in PanINs (Fig. 3d). Moreover, we found that semi-qRT-PCR was also sufficient to detect *KIAA1199* transcriptional expression, which might be due to the absence of *KIAA1199* transcripts in normal pancreatic cells (data not shown).

Because CA19-9, a widely used pancreatic cancer biomarker, is also elevated in pancreatitis^34^, we investigated whether KIAA1199 expression is induced by inflammation. We treated mice with caerulein, which mimics pancreatitis^34^. We found that caerulein-induced acute pancreatitis tissue did not express KIAA1199 (Fig. 3e).

To gain further insight into the kinetics of KIAA1199 expression during pancreatic tumorigenesis, we performed immunohistochemical analysis on human PanIN samples at different stages. KIAA1199 was expressed in both PanIN stage I and II samples (Fig. 3f and g, Supplementary Data 2, Supplementary Figure 2). These results suggest that KIAA1199 is specifically but heterogeneously expressed in PanIN lesions in a pancreatic cancer mouse model and in human samples.

### Detection of KIAA1199 Autoantibody from PanINs

**Figure 4:**
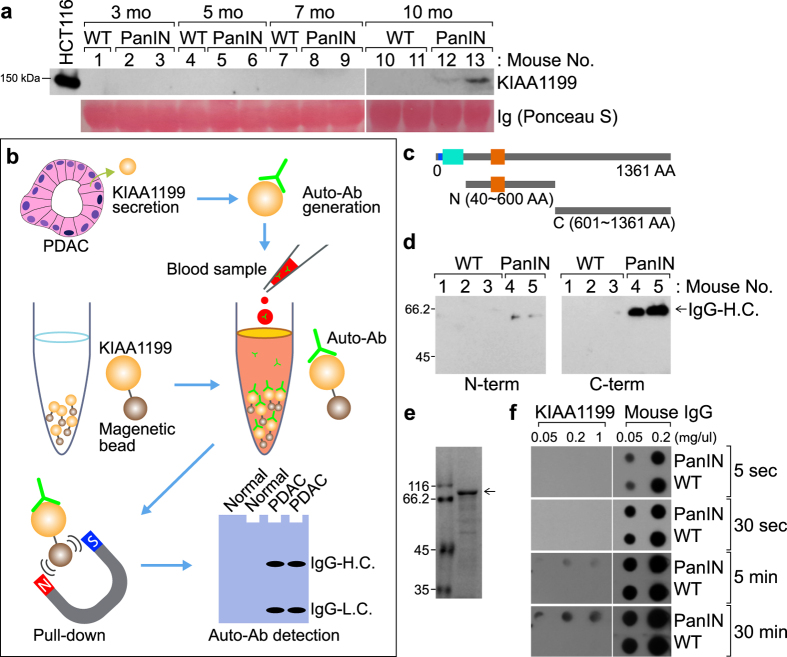
Detection of KIAA1199 autoantibody in mouse pancreatic intraepithelial neoplasia (PanIN). (**a**) Detection of KIAA1199 in mouse blood serum. Mouse blood sera from each group (3-, 5-, 7-, and 10-month-old wild-type [WT] or PanIN [*Pdx1-Cre:K-Ras*^*LSLG12D*^] mice) were analyzed by immunoblotting for KIAA1199. Immunoglobulin (Ig) served as an internal control. HCT116 colon cancer cells served as a positive control. (**b**) Experimental scheme of KIAA1199 autoantibody detection. Pancreatic ductal adenocarcinoma (PDAC) cells secrete KIAA1199 protein. Host immune system generates autoantibodies against KIAA1199. Blood samples were collected and incubated with KIAA1199 recombinant protein. KIAA1199 autoantibody binds to KIAA1199 recombinant protein. Immune complex is precipitated using magnetic beads. KIAA1199 autoantibody (IgG heavy chain [H.C.] and light chain [L.C.]) is detected by immunoblotting using antibody against the host. (**c**) Purification of KIAA1199 recombinant protein (N- and C-termini). (**d**) Detection of KIAA1199 autoantibody using immunoprecipitation. Mouse blood sera were immunoprecipitated using recombinant KIAA1199 proteins (N- and C-termini). Then, immunoprecipitates were analyzed by immunoblotting using mouse immunoglobulin G (IgG) conjugated with horseradish peroxidase (HRP). (**e**) Purification of GST-KIAA1199 protein C-terminus. GST-KIAA1199 (C-terminus) protein was purified using the bacterial protein expression system. Arrow indicates purified KIAA1199 protein (Coomassie staining). (**f**) Detection of KIAA1199 autoantibody using dot blot assays. Different exposure times are indicated. Recombinant KIAA1199 protein (C-terminus) and mouse IgG (a negative control) were transferred onto the membrane for dot blot assays. The sera of mice (WT or PanINs [*Pdx1-Cre:K-Ras*^*LSLG12D*^] mice; 5-month-old) were added to probe for the KIAA1199 autoantibody.

Because KIAA1199 is a secreted protein^31^ (see Fig. 2j), we hypothesized that KIAA1199 is secreted into the blood of PanIN mice. We collected blood samples from *Pdx1-Cre:K-Ras*^*LSLG12D*^ compound strains and quantified the expression of KIAA1199 protein. Consistent with immunostaining and qRT-PCR results (see Fig. 3b–d), immunoblotting analysis showed KIAA1199 protein expression in blood samples from PanIN mice (*Pdx1-Cre:K-Ras*^*LSLG12D*^) but not in normal pancreatic cells of WT mice (Fig. 4a). These results suggest that KIAA1199 is specifically expressed and secreted into the blood of PanIN mice.

We observed a difference in KIAA1199 expression detection sensitivity between immunoblotting analysis and qRT-PCR (see Figs 2 and 3). Unlike qRT-PCR, direct immunoblotting of blood samples only detected KIAA1199 expression in PanINs from 10-month-old *K-Ras* mouse models (Fig. 4a). This discrepancy might be due to the different sensitivity in detection methods we employed.

To overcome this technical limitation, we assessed the autoantibody against KIAA1199. Autoantibodies are generated by the host immune system^35^. Given the specific expression of KIAA1199 in human and mouse PDACs, we investigated whether KIAA1199 autoantibody can be detected using recombinant KIAA1199 protein as immobilized bait (Fig. 4b). We purified the N-terminus (40–600 amino acids) and C-terminus (601–1361 amino acids) of KIAA1199 protein using the *in vitro* transcribed and translated protein synthesis system (Fig. 4c). Then, we performed pull-down assays to precipitate KIAA1199 autoantibody from the blood serum of WT and PanINs mice. We used immunoblotting assays and detected immunoglobulin G (IgG) heavy chain of KIAA1199 autoantibody (Fig. 4d). Notably, the C-terminus of KIAA1199 protein showed an increase in immunoprecipitation with KIAA1199 autoantibody compared with the N-terminus of KIAA1199 protein (Fig. 4d, the right panel). Thus, we used the C-terminus of KIAA1199 for our subsequent detection approaches.

Next, we sought to improve the methods for detecting KIAA1199 autoantibody. Using a bacterial protein expression system, we purified the recombinant protein of KIAA1199 (C-terminus) (Fig. 4e). Then, we performed dot blot assays using KIAA1119 protein (C-terminus) and mouse IgG (a positive control). Dot blot results showed the specific detection of KIAA1199 autoantibody in 5-month-old mice bearing PanINs (Fig. 4f), whereas the secreted KIAA1199 protein is detectable in serum only from 10-month-old mice (see Fig. 4a). These results suggest that the KIAA1199 autoantibody is a sensitive method to assess the expression of KIAA1199 in PanIN mouse models.

### Detection of KIAA1199 Autoantibody in Human Pancreatic Adenocarcinoma

**Figure 5:**
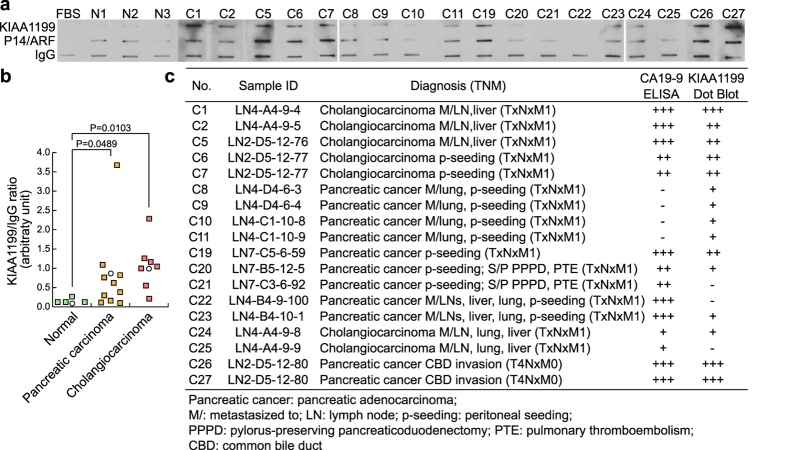
Detection of KIAA1199 autoantibody in patients with pancreatic cancer. (**a**) Detection of KIAA1199 autoantibody in the blood samples of human pancreatic cancer patients. The blood samples from healthy individuals (N1~N3) and patients with pancreatic adenocarcinoma or cholangiocarcinoma were incubated with immobilized proteins (KIAA1199, P14/ARF, and immunoglobulin G [IgG]). Then, blots were analyzed using anti-human IgG-horseradish peroxidase (HRP). Fetal bovine serum–treated samples served as a negative control. p14/ARF protein and IgG served as controls. Blot images shown have been cropped to show the relevant band. Full-length blots are presented in Supplementary Figure 3. (**b**) Pancreatic carcinoma and cholangiocarcinoma patient sample information. Enzyme-linked immunosorbent assay (ELISA) results of CA19-9 (data not shown) were added for comparison. (**c**) Statistical analysis of KIAA1199 autoantibody in pancreatic cancer. Dot blots were analyzed using ImageJ software. Statistical significance was determined using the Welch *t*-test.

Next, we sought to determine whether KIAA1199 autoantibody is also detectable in blood samples from patients with pancreatic cancer. We performed dot blot assays using purified recombinant protein of the C-terminus of KIAA1199. KIAA1199 protein was immobilized on nitrocellulose membranes and incubated with patient blood samples. Then, anti-human IgG conjugated with horseradish peroxidase was used to detect KIAA1199 autoantibody bound to KIAA1199 recombinant protein. KIAA1199 autoantibody was detected in blood samples of patients with pancreatic cancer (P = 0.0489; N = 10) but not in the blood samples from healthy individuals (N = 3) (Fig. 5a and b). We also detected KIAA199 autoantibody in cholangiocarcinoma samples (P = 0.0103; N = 6) (Fig. 5a and b). Interestingly, compared with the results of CA19-9 enzyme-linked immunosorbent assay, KIAA1199 autoantibody was detected in four pancreatic cancer samples (C8, C9, C10, and C11), whereas CA19-9 was not detected (Fig. 5c). Of note, KIAA1199 autoantibody was not found in two pancreatic cancer samples (C21 and C22) that expressed CA19-9 (Fig. 5c). These results suggest that detecting KIAA1199 autoantibody from patient blood samples is a sensitive method for assessing KIAA1199 expression in human pancreatic cancer.

## Discussion

KIAA1199 is expressed in the cytoplasm of epithelial cells and is secreted into the extracellular matrix^36^. Despite the implication of KIAA1199 in several human diseases including cancer, the molecular mechanism of KIAA1199 remains ambiguous. Dysregulation of KIAA1199 is associated with nonsyndromic deafness^26^ and rheumatoid arthritis^37^. Overexpression of KIAA1199 has been found in several human cancers. In gastric cancer, KIAA1119 is a potential prognostic and lymph node metastatic marker^30^. Moreover, KIAA1199 is highly expressed in adenoma, implying the potential application of KIAA1199 as a biomarker for early stages of cancer^38^.

Although CA19-9 is used as a prognostic biomarker for pancreatic cancer, not all pancreatic patients display elevated levels of CA19-9. Therefore, to avoid false-negative results, more specific biomarkers for diagnosis of early-stage pancreatic cancer are needed. Because KIAA1199 is upregulated and secreted in human pancreatic cancer cells, KIAA1199 could be a useful biomarker. We found that detection of autoantibody against KIAA1199 is more sensitive than the detection of KIAA1199 protein (Figs 3 and 4). In *K-Ras* PanIN mouse serum, the C-terminus of KIAA1199 protein is more sensitive for detecting KIAA1199 autoantibody than the N-terminus of KIAA1199 protein. Furthermore, by analyzing human patient tissues, we validated that KIAA1199 can be detected at PanIN stage I. Notably, KIAA1199 autoantibody was detected in metastatic pancreatic cancer samples for which the CA19-9 biomarker gave false-negative results (samples C8–C11), indicating the difference in the sensitivity between KIAA1199 and CA19-9. Whereas CA19-9 often has false-positive results in pancreatitis, we found that KIAA1199 was not expressed in the mouse pancreatitis model (see Fig. 3e). Therefore, the combination of KIAA1199 and CA19-9 may be useful for screening for early pancreatic cancer.

Intriguingly, KIAA1199 autoantibody was also detected in cholangiocarcinoma samples. Cholangiocarcinoma arises from the epithelial cells of the bile duct and is characterized by late diagnosis and poor survival^39^. Similar to CA19-9, it is also plausible that KIAA1199 expression might be highly correlated with epithelial cell–driven cancer.

Early detection of PanINs prior to development of invasive malignant pancreatic cancer would provide an opportunity to cure patients. However, PanINs (<5 mm) are not detectable even by recent high-resolution imaging methods. Therefore, reliable biomarkers are needed to detect the differentiating precursor lesions. Although our data suggest that KIAA1199 is a potential biomarker for pancreatic cancer, further study is needed to assess the sensitivity and specificity of KIAA1199 autoantibody in PanIN patient serum for early diagnosis.

Moreover, further research is needed to determine whether KIAA1199 can be used for prognosis, assessment of therapeutic response, or diagnosis of recurrence. Taken together, our results suggest that KIAA1199 autoantibody is a biomarker for early pancreatic cancer, which may complement current diagnostic methods.

## Methods

### Cell Culture

Human PDAC cell lines AsPC-1, BxPC-3, and Panc-1 were obtained from the American Type Culture Collection (ATCC, Manassas, VA). Cells were grown in Roswell Park Memorial Institute-1640 medium (Thermo Fisher Scientific, Waltham, MA) containing 10% fetal bovine serum and 1% penicillin/streptomycin at 37 °C in an incubator. Each cell line was tested and authenticated by ATCC.

### Oncomine Database Analysis

cDNA microarray datasets of pancreatic cancer and normal tissue samples were analyzed using the Oncomine database (www.oncomine.org). Our parameters included P < 0.0001, fold change >2, and the top 1% of ranked genes, as previously described^40^,^41^.

### Gene Expression Analysis

RNA was extracted by TRIzol (Invitrogen, Carlsbad, CA) and converted into cDNA using SuperScript II (Invitrogen) with random hexamers. For gene expression analysis, qRT-PCR was performed. qRT-PCR results were quantified by comparative 2^−ΔΔCt^ methods. For internal controls, *GAPDH, HPRT*, and 18s *rRNA* were used. The primer sequences used in these analyses are available upon request.

### Immunohistochemistry

Human pancreatic cancer and PanIN tissue microarrays were purchased from US Biomax (PA207, BIC14011a, PA242b; Rockville, MD) and were immunostained with anti-KIAA1199 antibody (Abcam, Cambridge, UK; Santa Cruz Biotechnology, Dallas, TX), as previously described^41^. For fluorescent immunohistochemistry, Alexa Fluor 488 or 555, fluorescence-conjugated secondary antibodies were used (Invitrogen). For chromogenic immunohistochemistry, horseradish peroxidase-conjugated secondary antibody and 3,3′- Diaminobenzidine substrate were used (Vector Laboratories, Burlingame, CA). Pancreatic tissue samples from *Pdx1-Cre:K-Ras*^*LSLG12D*^ and mice with caerulein-induced acute pancreatitis were collected and fixed with 10% formalin. After processing for paraffin embedding, sectioned samples were subjected to immunostaining or hematoxylin and eosin staining using standard protocols. Samples were mounted using ProLong Gold Antifade reagent (Invitrogen) or Permount (Fisher Scientific, Fair Lawn, NJ) and observed using a fluorescent microscope (Axio observer, Zeiss, Oberkochen, Germany; 63X magnification).

### Pancreatic Cancer Mouse Model

As previously described^40^, *Pdx1-Cre*:*K-Ras*^*LSLG12D*^ compound strains were generated by breeding each strain. All experimental protocols were approved by the Institutional Animal Care and Use Committee of The University of Texas MD Anderson Cancer Center, and experiments were carried out in accordance with approved guidelines.

### Caerulein-Induced Acute Pancreatitis Mouse Model

Caerulein was administered with six (1-h interval) intraperitoneal injections (50 μg/kg) into the mice (C57BL/6 J, 6-week-old, male). Phosphate-buffered saline solution was injected into mice as a negative control (vehicle). Mice were killed at one of two time points: 8 hours after caerulein injection (acute pancreatitis phase) or 4 days later (recovery phase). All mice were maintained and treated in compliance with Institutional Animal Care and Use Committee guidelines.

### Immunoblotting Assay

Proteins were obtained as previously described^42^, and the following antibodies were used for immunoblotting: KIAA1199 (Abcam and Santa Cruz Biotechnology) and tubulin (Sigma-Aldrich, St. Louis, MO).

### Detection of Autoantibody

For *in vitro* transcription and translation of KIAA1199 N-terminus and C-terminus protein, we utilized the TNT Quick Coupled Transcription/Translation system (Promega, Madison, WI). KIAA1199 recombinant protein was purified using the glutathione-S-transferase (GST) bacterial expression system. Briefly, the coding sequences of KIAA1199 were constructed into pGEX-6T bacterial expression plasmids. Then, the plasmids were transformed into BL21 *Escherichia coli*. After adding isopropyl β-D-1-thiogalactopyranoside (0.1 mM for 6 hours), we subjected cell lysates to protein purification. For autoantibody detection, mouse serum (3-, 5-, 7-, and 10-month-old wild-type [WT] or PanIN [*Pdx1-Cre:K-RasLSLG12D*] mice) was collected by centrifugation (5,000 rpm) from the blood samples. Serum (10 μL) was incubated with GST-recombinant protein. Then, precipitates were collected using magnetic beads and detected by immunoblotting using horseradish peroxidase–conjugated anti-mouse antibody and SuperSignal West Pico Chemiluminescent substrate (Thermo Fisher Scientific). For dot blot assays, purified KIAA1199 protein was transferred to nitrocellulose membranes for immunoblotting using standard procedures.

### Autoantibody Detection from Human Sera Samples

Human patient sera samples were collected after obtaining informed consent under a protocol approved by the Institutional Review Board at the Gyeongsang National University Hospital (Jinju, Republic of Korea). All experiments were carried out in accordance with approved guidelines. Human patient sera samples were obtained from the medical center of Gyeongsang National University. Recombinant proteins (KIAA1199: 1 μg/well; P14/ARF: 0.5 ng/well) and human IgG (0.02 ng/well, Santa Cruz Biotechnology) were immobilized on nitrocellulose membranes using the Bio-Dot SF Microfiltration apparatus (Bio-Rad Laboratories, Hercules, CA). Each membrane was incubated with diluted patient serum in blocking buffer (1:1,000 in 1% skim milk containing TBST) for 30 min. After washing with TBST, membranes were incubated with secondary antibody (goat anti-human IgG-horseradish peroxidase, 1:20,000 in blocking buffer) for 30 min. Reacted antibody was detected by ECL and x-ray film exposure and blots were analyzed using Image J software. CA19-9 was detected using an enzyme-linked immunosorbent assay kit (Calbiotech, Inc., Spring Valley, CA).

### Statistical Analysis

The Student *t*-test and Welch *t*-test were used for comparing two groups (n ≥ 3). P values less than 0.05 were considered significant. Error bars indicate standard deviation.

## Additional Information

**How to cite this article**: Suh, H. N. *et al*. Identification of KIAA1199 as a Biomarker for Pancreatic Intraepithelial Neoplasia. *Sci. Rep.* **6**, 38273; doi: 10.1038/srep38273 (2016).

**Publisher's note:** Springer Nature remains neutral with regard to jurisdictional claims in published maps and institutional affiliations.

## Supplementary Material

Supplementary Information

Supplementary Data 1

Supplementary Data 2
