# Supplementary material for: Identification of KIAA1199 as a Biomarker for Pancreatic Intraepithelial Neoplasia
Source: Sci Rep. 2016 Dec 6;6:38273. doi: 10.1038/srep38273 (PMC5138641; doi:10.1038/srep38273)
Supplement: Supplementary Information [file srep38273-s1.pdf]

Identification of KIAA1199 as a Biomarker for Pancreatic Intraepithelial Neoplasia

Han Na Suh,[1#] Sohee Jun,[1#] Ah-Young Oh,[2#] Mrinal Srivastava,[1] Sunhye Lee,[1] Cullen M. Taniguchi,[1] Songlin Zhang,[3] Won Sup Lee,[4] Junjie Chen,[1] Bum-Joon Park,[2] and Jae-Il Park[1,5,6,*]

[1]Department of Experimental Radiation Oncology, The University of Texas MD Anderson Cancer Center, Houston, TX 77030, USA

[2]Department of Molecular Biology, Pusan National University, Busan, Republic of Korea

[3]Department of Pathology, The University of Texas Medical School, Houston, TX 77030, USA.

[4]Department of Internal Medicine, Gyeong-Sang National University Hospital, Jinju, Republic of Korea

[5]Graduate School of Biomedical Sciences at Houston, Houston, TX 77030, USA

[6]Program in Cancer Biology, The University of Texas MD Anderson Cancer Center, Houston, TX 77030, USA

[#]These authors equally contributed to this work.

*Correspondence: Jae-Il Park

SUPPLEMENTARY INFORMATION

Supplementary Figures 1, 2, and 3

Supplementary Data 1 and 2

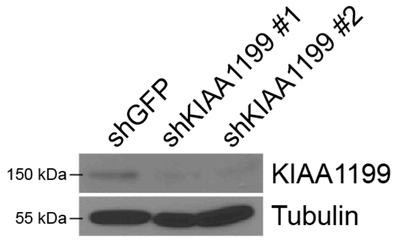

Supplementary Figure 1. Expression of KIAA1199 in Panc-1
Panc-1 was transfected with shGFP (negative control), shKIAA1199#1, or shKIAA1199#2 (endogenous depletion of specific for KIAA1199).

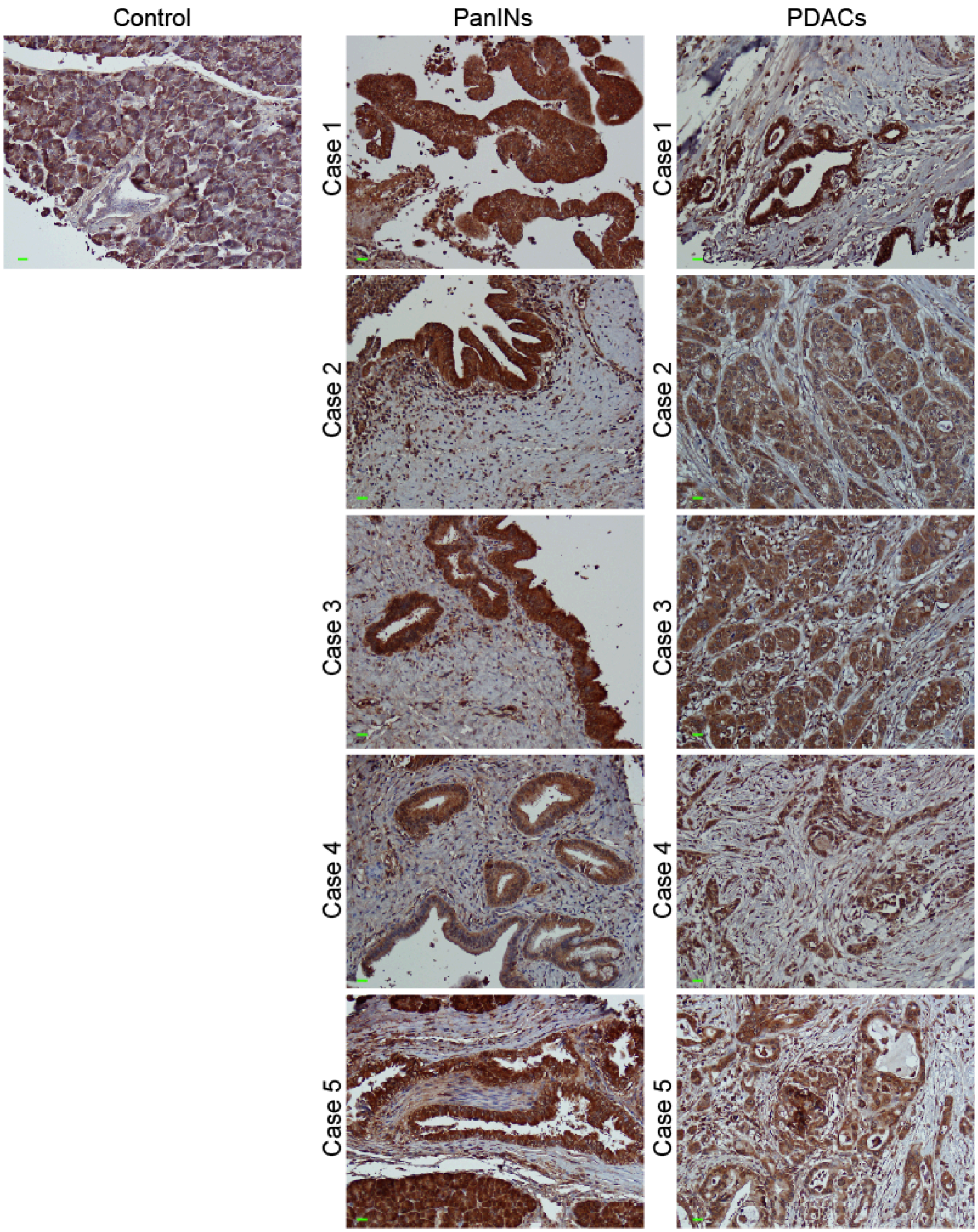

Supplementary Figure 2. Expression of KIAA1199 in PanINs and PDACs
TMA slides (BIC14011a and PA242b) were stained with KIAA1199 antibody (SantaCruz SC-164775, 1:25 dilution) and peroxidase-conjugated anti-rabbit 2nd antibody (Jackson Immunoresearch, 1:200 dilution). DAB substrate was used for chromogenic detection. Scale bars= 20 μm.

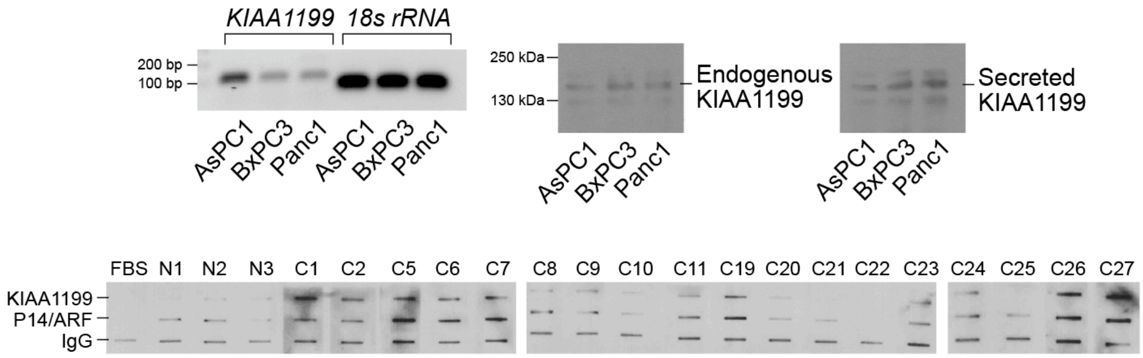

Supplementary Figure 3. Full-length gels and blots
Related images of Figure 2h-2j and Figure 5a

Supplementary Data 1. Oncomine analysis
List of two cDNA microarray datasets were analyzed using Oncomine (Gruntzmann [177 genes] and Pei [195 genes]). 21 genes were selected as the top 1% upregulated genes (fold change > 2; P < 0.0001).

Supplementary Data 2. KIAA1199 expression scoring in PanINs and PDAC (Biomax TMA BIC14011a and PA242b). KIAA1199 expression intensity was scored (no expression: –; expression: +1~+3 from low to high expression).
